# 

**Published:** 1857-09

**Authors:** 


*Proceedings of the Medical Society at the Meeting in Ottawa in May last.*

Sometime in June I received from the Secretary a copy of the Proceedings of the Lasalle County Medical Society, with a request that they should be published in the *Journal.* I accordingly gave the manuscript to the printer, to be inserted in the number for July. As more manuscript was given to the printer than was required to fill that number of the *Journal,* this, together with one or two other short articles, was omitted and returned with the proof sheets. I again sent the Ottawa communication to the printer, with instructions to insert it in the number for August. When that number was made up, however, the article was again found missing, and I have not been able to find it since. The communication was short, occupying but one sheet of letter-paper, and might have been lost by the messenger in taking it to the printing office. The only matter of importance which it contained was the fact that Dr. D. D. Thompson, of Ottawa, had united with a Homœopathic practitioner of that city in business, and in consequence thereof had been expelled from the Medical Society. On the other hand, another physician, whose name we do not remember, who had been practising the "*little pill*" system, publicly renounced it and joined the regular society. I hope this explanation will be satisfactory to our friends in Ottawa; and if they send any more communications for the *Journal* I will see that they are properly taken care of.

